# Effect of a Structured Multilevel Telehealth Service on Hospital Admissions and Mortality During COVID-19 in a Resource-Limited Region in Brazil: Retrospective Cohort Study

**DOI:** 10.2196/48464

**Published:** 2024-06-10

**Authors:** Clara Rodrigues Alves Oliveira, Magda Carvalho Pires, Karina Cardoso Meira, Jordana Cristina de Jesus, Isabela Nascimento Borges, Maria Cristina Paixão, Mayara Santos Mendes, Leonardo Bonisson Ribeiro, Milena Soriano Marcolino, Maria Beatriz Moreira Alkmim, Antonio Luiz Pinho Ribeiro

**Affiliations:** 1 Department of Internal Medicine Medical School Universidade Federal de Minas Gerais Belo Horizonte Brazil; 2 Telehealth Center University Hospital and Telehealth Network of Minas Gerais Universidade Federal de Minas Gerais Belo Horizonte Brazil; 3 Department of Statistics Universidade Federal de Minas Gerais Belo Horizonte Brazil; 4 Health School Universidade Federal do Rio Grande do Norte Natal Brazil; 5 Institute for Health Technology Assessment Porto Alegre Brazil

**Keywords:** COVID-19, telehealth, health care, clinical outcomes, hospital admission, mortality, adoption, effectiveness, digital health tool, flu, teleconsultation, digital health, digital literacy, telemonitoring

## Abstract

**Background:**

The COVID-19 pandemic represented a great stimulus for the adoption of telehealth and many initiatives in this field have emerged worldwide. However, despite this massive growth, data addressing the effectiveness of telehealth with respect to clinical outcomes remain scarce.

**Objective:**

The aim of this study was to evaluate the impact of the adoption of a structured multilevel telehealth service on hospital admissions during the acute illness course and the mortality of adult patients with flu syndrome in the context of the COVID-19 pandemic.

**Methods:**

A retrospective cohort study was performed in two Brazilian cities where a public COVID-19 telehealth service (TeleCOVID-MG) was deployed. TeleCOVID-MG was a structured multilevel telehealth service, including (1) first response and risk stratification through a chatbot software or phone call center, (2) teleconsultations with nurses and medical doctors, and (3) a telemonitoring system. For this analysis, we included data of adult patients registered in the Flu Syndrome notification databases who were diagnosed with flu syndrome between June 1, 2020, and May 31, 2021. The exposed group comprised patients with flu syndrome who used TeleCOVID-MG at least once during the illness course and the control group comprised patients who did not use this telehealth service during the respiratory illness course. Sociodemographic characteristics, comorbidities, and clinical outcomes data were extracted from the Brazilian official databases for flu syndrome, Severe Acute Respiratory Syndrome (due to any respiratory virus), and mortality. Models for the clinical outcomes were estimated by logistic regression.

**Results:**

The final study population comprised 82,182 adult patients with a valid registry in the Flu Syndrome notification system. When compared to patients who did not use the service (n=67,689, 82.4%), patients supported by TeleCOVID-MG (n=14,493, 17.6%) had a lower chance of hospitalization during the acute respiratory illness course, even after adjusting for sociodemographic characteristics and underlying medical conditions (odds ratio [OR] 0.82, 95% CI 0.71-0.94; *P*=.005). No difference in mortality was observed between groups (OR 0.99, 95% CI 0.86-1.12; *P*=.83).

**Conclusions:**

A telehealth service applied on a large scale in a limited-resource region to tackle COVID-19 was related to reduced hospitalizations without increasing the mortality rate. Quality health care using inexpensive and readily available telehealth and digital health tools may be delivered in areas with limited resources and should be considered as a potential and valuable health care strategy. The success of a telehealth initiative relies on a partnership between the involved stakeholders to define the roles and responsibilities; set an alignment between the different modalities and levels of health care; and address the usual drawbacks related to the implementation process, such as infrastructure and accessibility issues.

## Introduction

The COVID-19 pandemic has accelerated the adoption of telehealth in outpatient settings [1]. The demands generated by the pandemic, such as the need for social distancing and home isolation in an effort to reduce virus spread and infection rates, to decrease the pressure on overwhelmed health systems, and to increase health care providers’ safety, determined the scaled-up use of telehealth tools worldwide [2].

Taking these new challenges into account, the Telehealth Network of Minas Gerais (TNMG), one of the largest public telehealth services in Latin America, developed a synchronous teleconsultation and telemonitoring service to assist patients with suspected or confirmed COVID-19 [3,4]. This service, named TeleCOVID-MG, was first deployed in two Brazilian medium-sized cities and was then expanded to assist the entire community of Universidade Federal de Minas Gerais (UFMG), a large public Brazilian university [3].

Although many telehealth services were developed and implemented during the pandemic, data addressing the clinical effectiveness of the use of a telehealth service for the health assistance of patients with respiratory symptoms are scarce [5,6]. Most of the available studies have focused on different outcomes such as user satisfaction, system usability, and emotional comfort [3,5]. However, there is a lack of studies analyzing the impact of telehealth services on more objective outcomes such as hospitalization and mortality rates compared to standard health care support, which is usually delivered in an in-person manner. Moreover, the studies that do exist in this regard often include small sample sizes and report conflicting results [6].

Casariego-Vales et al [7] conducted a study to assess the effectiveness of a proactive telemonitoring approach for patients with COVID-19. The study was conducted in the northwest region of Spain during the third wave of the pandemic. The results demonstrated that the patients who were enrolled in a systematic telemonitoring program, which included different intensities of approaches tailored to the patient’s risk for adverse outcomes, presented lower rates of emergency department visits and hospitalization as compared to those of the patients who did not participate in this systematic telemonitoring program. Additionally, these systematically telemonitored patients presented shorter hospital stays and a lower mortality rate during their first hospitalization [7].

Another study examined the impact of a telehealth intervention in Michigan, United States, which was a nurse-led, telephone-based active management protocol for individuals with COVID-19 who were in home isolation. Although the intervention group showed a lower rate of hospitalization within 30 days compared to that of the control group, this result was not statistically significant [8].

To obtain additional information on the effects of telehealth adoption with respect to clinical outcomes, the main goal of this study was to evaluate the impact of the adoption of a structured multilevel telehealth service, including teleconsultation and telemonitoring services and digital health tools, on relevant clinical outcomes such as hospital admission and mortality. Toward this end, we compared the outcomes presented by the patients with flu syndrome who did and did not use the telehealth service during the respiratory illness course in a limited-resource region in Brazil where TeleCOVID-MG was deployed.

## Methods

### The TeleCOVID-MG Service

TeleCOVID-MG was developed by the TNMG, which represents a telehealth network created in 2005 through a partnership among seven public universities of Minas Gerais State in the southeast region of Brazil. The TNMG is a large telehealth service in Brazil and develops different activities in the areas of clinical support, research, and tele-education [9].

Soon after the first case of COVID-19 was confirmed in Brazil, the multidisciplinary team of the TNMG, including physicians with expertise in telemedicine, infectious diseases specialists, nurses, managers, and information technology specialists, started to develop the TeleCOVID-MG system, according to the issued World Health Organization and Brazilian Ministry of Health Guidelines for COVID-19. Based on these guidelines and other available scientific evidence about COVID-19, instructional material was developed for training the TeleCOVID-MG team. As new scientific evidence emerged, the system and the instructional material were updated. TeleCOVID-MG was designed as a structured multilevel telehealth service, including teleconsultation and telemonitoring services and digital health tools (ie, a chatbot) [10]. TeleCOVID-MG software runs on a web environment, which allows the full recording of all activities taking place on the platform [3,4]. The TeleCOVID-MG structured database and the data collected in the software were used for this study.

The service was first implemented in two Brazilian cities, Divinópolis and Teófilo Otoni, in May 2020. The service was then expanded in November 2020 to assist the faculty, public servants, and students of the UFMG. The service provided health support for the populations of Divinópolis and Teófilo Otoni until December 2021 and for the UFMG community until March 2023. TeleCOVID-MG was offered to the entire populations of these cities and the users were not charged for using it.

TeleCOVID-MG was built to work through the intersection of four different levels, as described elsewhere [3]. In brief, level 1 represents the user’s gateway to the service, which is achieved via a chatbot developed by the TNMG team or through a special telephone call center [10]; level 2 represents the nursing staff; level 3 represents the medical staff; and level 4 represents the telemonitoring service, conducted by medical students under supervision. Every health professional and medical student in the program was extensively trained to use the TeleCOVID-MG system and to provide health assistance to the patients, as previously described [3,4].

At level 1, the screening and risk stratification of the patient with respiratory complaints was conducted by the chatbot software or by professionals of the call center who were trained for the task, which was performed through a list of questions that were established by drawing upon the best available evidence [10]. According to the severity of the symptoms and the comorbidities, the patient was sent to teleconsultation with the nursing staff, at level 2, or with the medical staff, at level 3. At the end of this teleconsultation, the health professional could advise the patient to adhere to home isolation or to seek onsite evaluation at a primary care center or an emergency unit. In addition to carrying out teleconsultations, the TeleCOVID-MG system allowed the health professionals to issue prescriptions, reports, and orders for diagnostic COVID-19 tests. All of these documents could be easily downloaded by the users. The software also enabled the generation of the compulsory report of suspected or confirmed COVID-19 cases, in compliance with requirements of the Brazilian Health Ministry [3,4].

All patients who were assessed by the nursing or medical teams at levels 2 and 3 were included in the telemonitoring program, which provided support for at least 10 days after the onset of respiratory symptoms. This telemonitoring service was delivered through a phone call; the patients with no alarm signs or decompensated comorbidities were monitored every 48 hours, while the patients with alarm signs and/or decompensated comorbidities were monitored every 24 hours. Undergraduate medical students from the local universities working under the supervision of a physician or a nurse composed the telemonitoring team [3,4].

There was also a nurse or a physician available to supervise and offer support to the level 2 and level 3 professionals, respectively. All of the supervisors were trained for the task and were available for clinical discussions and doubts clarification throughout the teleconsultation and telemonitoring duties. It is important to emphasize that, according to the clinical course of illness presented by the patient, a professional of any level, at any time, might request an evaluation by the staff of another level.

Finally, throughout the period that TeleCOVID-MG was in operation, a periodic and open dialogue was maintained with the local health managers to define the roles and responsibilities of the stakeholders and to set an alignment between the different modalities and levels of health care.

### Study Design and Procedure

A retrospective cohort study was performed with adult patients (≥18 years old) who were registered in the Divinópolis and Teófilo Otoni Flu Syndrome compulsory notification databases between June 1, 2020, and May 31, 2021. The Divinópolis and Teófilo Otoni Flu Syndrome databases include patients who live and/or have received health support during the illness course in one of these two cities.

The exposed group (TeleCOVID-MG group) was composed of the registered patients with flu syndrome who used TeleCOVID-MG at least once during their illness course and the unexposed group (control group) was composed of patients who did not use this telehealth service during the respiratory illness course. The main outcomes investigated were hospital admissions during the acute respiratory illness course and mortality.

Divinópolis and Teófilo Otoni are both medium-sized cities. However, Divinópolis has a higher demographic density and Human Development Index, and better socioeconomic, educational, and health indicators compared to those of Teófilo Otoni (Table 1) [11,12].

**Table 1:** Sociodemographic, economic, education, and health indicators of Divinópolis and Teófilo Otoni compared to Brazil’s overall indicators [11,12].

Indicators		Divinópolis	Teófilo Otoni	Brazil
**Sociodemographic indicators**				
	Population in 2022, n	231,091	137,418	203,062,512
	Demographic density (inhabitants/km^2^) in 2022	326.35	42.38	23.86
	Human Development Index in 2010	0.764	0.701	0.760
**Economic indicators**				
	Gross Domestic Product (Brazilian Real^a^) in 2020	29,331.04	19,873.45	35,935.74
	Employed population rate in 2020, %	27.4	22.1	28.4
	Average monthly salary of formal workers in minimum-wage jobs (Brazilian Real^a^) for 2021	2.1	1.8	2.3
**Education indicators**				
	Schooling rate from 6 to 14 years old in 2010, %	98.6	96.6	97.9
	Basic Education Development Index for the early years of elementary school in 2021	6.5	5.5	5.5
	**Health indicators**			
	Infant mortality rate (per 1000 live births) for 2020	9.13	13.66	11.20
	Number of physicians per 1000 inhabitants for 2022	3.16	1.97	2.38
	Households with sanitary sewage for 2010, %	90.1	77.1	63.2

^a^US $1=4.93 Real for June 2020.

### Data Acquisition

The data used in the analysis were extracted from four different databases: (1) the TeleCOVID-MG database, (2) Flu Syndrome compulsory notification database, (3) Severe Acute Respiratory Syndrome (SARS) compulsory notification database, and (4) Mortality Information System (MIS) database. Although the latter three are public databases, access to the identified data of the Flu Syndrome and SARS databases was obtained upon request to the municipal governments. In contrast, access to the identified data of the MIS database was solicited from the federal government.

The Flu Syndrome database includes data for the compulsory notification of patients with flu syndrome, the SARS database is composed of the compulsory notification of patients who were admitted to the hospital with SARS, and the MIS database is composed of the compulsory registration of the deaths of Brazilian citizens. Flu syndrome is defined by the Brazilian Health Ministry as the occurrence of an acute respiratory illness characterized by the presence of at least two of the following symptoms: fever, chills, sore throat, headache, cough, runny nose, and smell or taste disturbances. SARS is defined by the Brazilian Health Ministry as the occurrence of flu syndrome and at least one of the following symptoms: oxygen saturation below 95% (room air), dyspnea, persistent pressure or pain in the chest, and cyanosis of the lips or face [13].

It is important to note that the notification of flu syndrome and SARS can be made by any health professional at any level of health care. The patient’s data necessary for completing all required notification documents are standardized, but not all the fields are mandatory.

Data regarding hospital admission due to acute respiratory illness were obtained from the SARS database. To obtain more accurate mortality data, this register was extracted from all three public databases. Identifying the patient’s death in at least one of these databases was sufficient to characterize the final clinical outcome as “death.” For the mortality analysis, all the deaths that occurred throughout the study period were considered, regardless of their primary causes.

Potential cofounders used as adjustment variables included city of flu syndrome notification; age; sex; and underlying medical conditions such as chronic respiratory diseases, chronic cardiovascular diseases, diabetes, chronic kidney disease, immunosuppression, high-risk pregnancy, and chromosomal diseases. These data were extracted from the Flu Syndrome database. Finally, the linkage between the four databases was made using the patient’s name and their individual taxpayer register number.

This manuscript was written according to the STROBE (Strengthening the Reporting of Observational Studies in Epidemiology) guidelines for reporting observational studies [14].

### Statistical Analysis

Statistical analyses were performed in three steps: (1) descriptive analysis, (2) bivariate analysis (ie, evaluation of the association of the outcome with each variable of interest), and (3) multivariate analysis.

Descriptive analyses were run to summarize all variables, stratified into exposed and unexposed groups. Categorical variables (sex, city of flu syndrome notification, city of residence, underlying medical conditions, and clinical outcomes) are summarized by absolute and relative frequencies. The Shapiro-Wilk normality test was performed to determine whether the continuous variable (age) was normally distributed. This variable was found to have a nonnormal distribution and is therefore summarized using the median and IQR. There were no missing variables included in this analysis.

In the bivariate analysis, sociodemographic characteristics, city of flu syndrome notification, underlying medical conditions, and clinical outcomes were assessed using the *χ*^2^ test to compare proportions. The Wilcoxon rank sum test was used to compare medians of continuous variables.

Models for the primary outcomes were estimated by logistic regression; the shaping process of the prediction models divided variables into four blocks by adopting a forward approach, mutually inserted in regression models 1-4. As the primary goal of the analysis was to identify the association of the TeleCOVID-MG service with the clinical outcomes (mortality and hospital admissions during the acute respiratory illness course), this variable was tested in all four models. Model 1 included use of the TeleCOVID-MG service only, model 2 added the city of the flu syndrome notification, model 3 added sex and age, and model 4 added the underlying medical conditions. For the regression models, odds ratios (ORs) and their respective 95% CIs were estimated.

Concerning the choice of variables, we considered the recommendations of Steyerberg [15] and Harrell [16]. According to these authors, it is better to use subject matter knowledge than statistical methods for variable selection. In this strategy, significance testing of adjustment variables is not necessary, especially if subject-specific knowledge supports the estimated effects [14,15]. Thus, we grouped variables parsimoniously in blocks, aggregating variables more distal to those that are more proximal: geography (cities), biological features (age and sex), and clinical predisposing features (underlying medical conditions).

All analyses were performed in R software (version 4.0.2) with the tidyverse, lubridate, stringi, rlang, jsonlite, Rcurl, writexl, openxlsx, readxl, and lmtest packages. A *P* value <.05 was considered statistically significant.

### Ethical Considerations

Ethical approval for this study was obtained from the UFMG Research Ethics Committee (CAAE: 35953620.9.0000.5149). Informed consent was provided by all the patients who were supported by the TeleCOVID-MG service. The researchers signed a confidentiality term to access the identified data of the public databases. After the linkage of the databases, data were deidentified for the analysis. No compensation was provided to the research participants.

## Results

**Figure 1:**
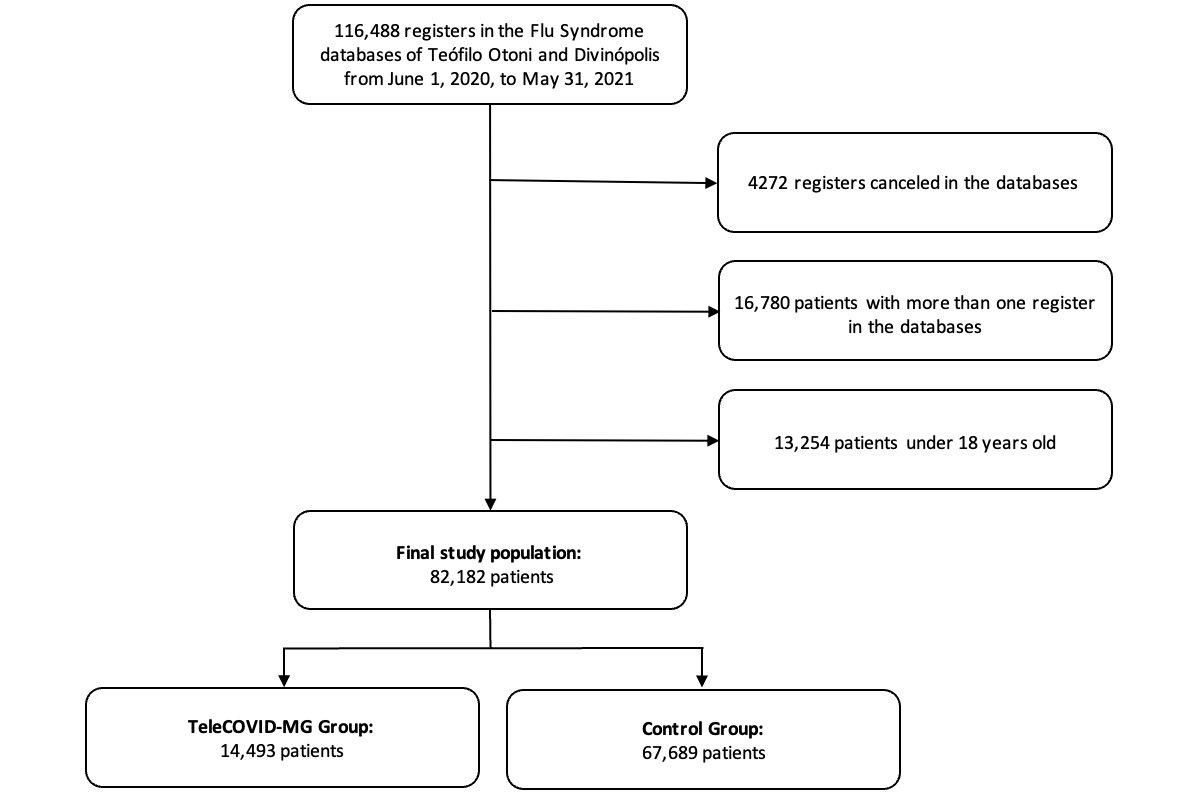
Flowchart of patient inclusion.

Throughout the study period, 116,488 registers were found in the Flu Syndrome notification systems of Teófilo Otoni and Divinópolis. Among these, 4272 registers were canceled in the database, 16,780 patients had more than one notification in the register, and 13,254 patients were under 18 years old. Therefore, the final study population was composed of 82,182 patients (Figure 1). For the analysis, only the first register of the patient in the Flu Syndrome database was used and none of the included variables contained missing data.

Among the 82,182 included patients, 14,493 (17.6%) used the TeleCOVID-MG service at least one time during the acute illness course (TeleCOVID-MG group) and the other 67,689 (82.4%) patients did not receive any kind of telehealth support by the TeleCOVID-MG service (control group). The sociodemographic and clinical characteristics of the patients are summarized in Table 2.

The TeleCOVID-MG group had a median age 2 years lower than that of the control group; a higher frequency of women; and slightly but significantly higher frequencies of patients with diabetes, chronic respiratory and cardiac diseases, and high-risk pregnancy (Table 2).

With regard to outcomes, the TeleCOVID-MG group had lower frequencies of both hospital admission (*P*<.001) and mortality (*P*=.003) compared to those of the control group (Table 2).

Among the 14,493 included patients who were accessed through the TeleCOVID-MG system during the illness course, 290 (2.0%) were advised to seek an onsite evaluation at a primary care center and 594 (4.1%) at an emergency department. The other 13,609 (93.9%) patients were advised to maintain domiciliary isolation and were monitored by the TeleCOVID-MG team.

In the regression analysis for the outcome hospital admission during the acute illness course, the telehealth intervention remained associated with a lower chance of hospitalization after adjusting for sociodemographic variables and underlying medical conditions (Table 3). In the fully adjusted model (model 4), there was a lower chance of hospitalization in patients who used the TeleCOVID-MG service, and a greater chance of this outcome was detected among the patients for whom the flu syndrome notification was reported in Teófilo Otoni, male patients, and patients with the following diseases: chronic cardiovascular disease, diabetes, chronic kidney disease, immunosuppression, and high-risk pregnancy (Table 3).

In the regression analysis for mortality (Table 4), the telehealth support was associated with a reduction in the chance of death in the model with the telehealth intervention alone (model 1) and in model 2 that included the city of flu syndrome notification. However, in models 3 and 4, which included sex, age, and underlying medical conditions, the OR values were close to 1.0. After the inclusion of all variables (model 4), age; Teófilo Otoni as the city of flu syndrome notification; male sex; and having chronic cardiovascular diseases, diabetes, immunosuppression, and chromosomal diseases remained associated with a greater chance of death due to flu syndrome (Table 4).

**Table 2:** Sociodemographic and clinical characteristics of the patients.

Characteristics			Overall (N=82,182)		TeleCOVID-MG group (n=14,493)		Control group (n=67,689)		*P* value^a^	
Age (years), median (IQR)			38 (28-51)		37 (27-49)		39 (29-51)		<.001	
Women, n (%)			44,847 (54.6)		8854 (61.1)		35,993 (53.2)		<.001	
**City of flu syndrome notification, n (%)**										<.001
	Divinópolis	46,313 (56.4)		3662 (25.3)		42,651 (63.0)				
	Teófilo Otoni	25,276 (30.8)		6269 (43.3)		19,007 (28.1)				
	Other	10,593 (12.9)		4562 (31.5)		6031 (8.9)				
**City of residence, n (%)**										<.001
	Divinópolis	47,507 (57.8)		6091 (42.0)		41,416 (61.2)				
	Teofilo Otoni	27,232 (33.1)		8205 (56.6)		19,027 (28.1)				
	Other	7443 (9.1)		197 (1.4)		7246 (10.7)				
**Underlying medical conditions, n (%)**										
	Chronic respiratory diseases	1272 (1.5)		253 (1.7)		1019 (1.5)		.03		
	Chronic cardiovascular diseases	2794 (3.4)		581 (4.0)		2213 (3.3)		<.001		
	Diabetes	2107 (2.6)		448 (3.1)		1659 (2.5)		<.001		
	Chronic kidney disease	203 (0.2)		20 (0.1)		183 (0.3)		.004		
	Immunosuppression	431 (0.5)		82 (0.6)		349 (0.5)		.45		
	High-risk pregnancy	407 (0.5)		103 (0.7)		304 (0.4)		<.001		
	Chromosomal diseases	139 (0.2)		28 (0.2)		111 (0.2)		.44		
**Clinical outcomes, n (%)**										
	Hospital admission	1980 (2.4)		269 (1.9)		1711 (2.5)		<.001		
	Death	1978 (2.4)		299 (2.1)		1679 (2.5)		.003		

^a^*P* values are based on the Pearson *χ*^2^ test for categorical variables or the Wilcoxon rank sum test for the continuous variable (age).

**Table 3:** Predictors of hospital admission according to logistic regression models.

Model			OR^a^ (95% CI)		*P* value
**Model 1**					
	TeleCOVID-MG group	0.73 (0.64-0.83)		<.001	
**Model 2**					
	TeleCOVID-MG group	0.64 (0.56-0.73)		<.001	
	City of flu syndrome notification (Teófilo Otoni)	1.58 (1.44-1.73)		<.001	
**Model 3**					
	TeleCOVID-MG group	0.85 (0.74-0.97)		.02	
	City of flu syndrome notification (Teófilo Otoni)	1.42 (1.29-1.56)		<.001	
	Male sex	1.75 (1.59-1.92)		<.001	
	Age	1.07 (1.07-1.08)		<.001	
**Model 4**					
	TeleCOVID-MG group	0.82 (0.71-0.94)		.005	
	City of flu syndrome notification (Teófilo Otoni)	1.50 (1.36-1.65)		<.001	
	Male sex	1.77 (1.61-1.95)		<.001	
	Age	1.07 (1.07-1.07)		<.001	
	Chronic respiratory diseases	1.14 (0.85-1.52)		.37	
	Chronic cardiovascular diseases	1.35 (1.15-1.58)		<.001	
	Diabetes	1.83 (1.54-2.17)		<.001	
	Chronic kidney disease	2.18 (1.34-3.43)		.001	
	Immunosuppression	1.87 (1.25-2.73)		.002	
	High-risk pregnancy	3.32 (1.29-6.96)		.005	
	Chromosomal diseases	1.33 (0.63-2.54)		.42	

^a^OR: odds ratio.

**Table 4:** Predictors of mortality according to logistic regression models.

Model			OR^a^ (95% CI)		*P* value
**Model 1**					
	TeleCOVID-MG group	0.83 (0.73-0.94)		.003	
**Model 2**					
	TeleCOVID-MG group	0.79 (0.69-0.89)		<.001	
	City of flu syndrome notification (Teófilo Otoni)	1.2 (1.09-1.32)		<.001	
**Model 3**					
	TeleCOVID-MG group	1.01 (0.89-1.15)		.85	
	City of flu syndrome notification (Teófilo Otoni)	1.07 (0.97-1.18)		.16	
	Male sex	1.5 (1.37-1.65)		<.001	
	Age	1.07 (1.06-1.07)		<.001	
**Model 4**					
	TeleCOVID-MG group	0.99 (0.86-1.12)		.83	
	City of flu syndrome notification (Teófilo Otoni)	1.12 (1.02-1.24)		.02	
	Male sex	1.52 (1.38-1.66)		<.001	
	Age	1.06 (1.06-1.07)		<.001	
	Chronic respiratory diseases	1.28 (0.96-1.67)		.08	
	Chronic cardiovascular diseases	1.34 (1.14-1.57)		<.001	
	Diabetes	1.51 (1.26-1.8)		<.001	
	Chronic kidney disease	2.28 (1.41-3.54)		<.001	
	Immunosuppression	2.37 (1.63-3.34)		<.001	
	High-risk pregnancy	2.02 (0.71-4.45)		.13	
	Chromosomal diseases	2.37 (1.28-4.1)		.004	

^a^OR: odds ratio.

## Discussion

### Principal Results

In an effort to avoid virus spread, preserve home isolation, and protect health care professionals from potential SARS-CoV-2 contamination, multiple telehealth initiatives emerged worldwide [1,17,18]. Along with the large adoption of telehealth tools in the pandemic context, concerns about usability, safety, costs, and efficacy related to this innovative practice have emerged. In this retrospective cohort study, we investigated the impact of the adoption of a structured multilevel public COVID-19 telehealth service on hospital admissions and mortality compared to the outcomes presented by patients who did not use the studied telehealth service. The results demonstrated a lower chance of hospitalization during the acute illness course in the TeleCOVID-MG group, even after adjusting for sociodemographic characteristics and underlying medical conditions, whereas there was no difference in mortality between the two groups.

The COVID-19 pandemic brought about an extra challenge for providing quality health assistance, which was particularly important for countries such as Brazil characterized by a large land area and significant health inequities [9,19]. In such regions, telehealth solutions offer even more advantages; thus, establishing that remote health services have the potential to provide high-quality health assistance will provide valuable information for health managers to better plan health interventions.

In addition to the clinical outcomes attained using the structured multilevel public COVID-19 telehealth service, it is reasonable to consider that this telehealth initiative contributed to achieving other important outcomes that were not measured in this study. In providing remote health support to the patients who did not need to seek an on-site evaluation in most situations, the telehealth service likely contributed to a decrease in contamination rates and thus an increase in health professionals’ safety, which are also important outcomes that should be considered in future research in this field.

There are various issues regarding the deployment of a telehealth service that must be carefully examined, including costs, professional and patient acceptance and satisfaction, technology availability, and data safety. Regarding costs, there is a large body of evidence demonstrating that telehealth initiatives can be a cost-saving option in different scenarios. Specifically in the context of COVID-19, our group has already demonstrated that the TeleCOVID-MG service increased the access to health care and was an economically attractive strategy [20-24]. Regarding the acceptance and availability of the technology, it is important to remark that teleconsultation was not regulated in Brazil before March 2020 and TeleCOVID-MG was a pioneer in providing this type of service to the population [25]. Approximately 17.6% of the adult population with respiratory symptoms in the two studied cities used the service during the analyzed period; in contrast to our initial concerns about patient acceptance, we consider this use rate to represent a promising kickoff of this initiative. Compared to other telehealth services described in the literature that reported higher use rates [26], this COVID-19 teleconsultation service was offered in a medium-income country for a population with a low digital literacy level and no culture of using telehealth services. Therefore, the results of this study provide an important demonstration that, in specific scenarios, it is possible to offer high-quality health care using inexpensive and readily available technology in areas with limited resources and low digital literacy. However, it is important to note that the success of a telehealth initiative in achieving good adoption and positive clinical results also relies on the agreements made with the local health managers and on the integration of this modality of care with existing in-person health care at different levels. Maintaining a partnership is essential to solve the usual drawbacks associated with implementation of a telehealth program, such as infrastructure and accessibility issues, and to renegotiate the roles and responsibilities of the stakeholders whenever necessary.

Finally, with the increasing use of technology worldwide, the use of telehealth tools is becoming more feasible, and legislations in this regard are being reviewed or created along with growing evidence about professional and user acceptance and usability [26-29]. It seems that the use of telehealth tools is occurring in a one-way direction, without return. Therefore, scientific efforts are needed to clarify their broader potential and limitations.

### Comparison With Prior Work

A large amount of evidence in different fields of telehealth has been produced; however, data regarding the clinical efficacy of this practice in the context of COVID-19 remain scarce [5,6].

A systematic review of 64 studies focusing on telehealth-based services for COVID-19 [5] revealed that only two studies measured mortality as an outcome and neither of these studies included control groups in their designs [30,31]. Another systematic review prepared by the Johns Hopkins University Center for Evidence-Based Practice evaluated the use of telehealth during the pandemic in different settings, but no comparative studies on the effectiveness of COVID-19 telehealth programs in reducing mortality were available [6].

Only two studies are available regarding the comparison of hospitalization rates between patients who were assisted by a specific COVID-19 telehealth program during the illness course and patients who did not receive telehealth support, with conflicting results [7,8]. Considering the sample sizes of both studies and the follow-up duration, the conclusion of the systematic review was that among the patients who received care for COVID-19, those who received an initial telehealth visit might have higher hospitalization rates compared with those of patients who received only in-person care (strength of evidence: low). This statement is open for discussion, as the primary study responsible for this conclusion in the systematic review presented additional information and results [7]. Although the primary study indicated that a high-intensity and protocolized telemonitoring program is related to higher hospitalization rates, this result was predictable given that the intensity of the telemonitoring actions was tailored to the risk of the patients in presenting adverse outcomes. Conversely, when considering all patients included in the study, the comparison between the groups showed that patients who were involved in a systematic telemonitoring program, which was tailored to the age and clinical characteristics of the patients, presented lower rates of hospital admissions than those of the patients who did not undergo the systematic telemonitoring program for COVID-19. Our results are consistent with the findings of this previous study, since we also demonstrated that engaging in systematic telehealth support provided through widely available technology can lead to positive clinical outcomes. Thus, our results further underscore the potential of the use systematic telehealth support as part of a comprehensive health care strategy.

It is worth emphasizing that both studies included in the systematic review [7,8] were conducted in high-income countries and involved relatively short follow-up (68 days and 30 days). In contrast, we evaluated the clinical effectiveness of a structured multilevel public COVID-19 telehealth service in a limited-resource region and included 82,182 patients who were followed up for 1 year. In this pioneering analysis, which spans an extended duration and involves a significant number of patients, the findings highlighted that embracing a public COVID-19 telehealth service yielded a positive impact with respect to hospital admissions without increasing mortality.

### Limitations

The most important limitation of this study is related to the data quality of the public databases used in the analysis, mainly regarding the comorbidities, socioeconomic status, and ethnicity of the included patients.

Regarding the comorbidities, although these data were not missing, there is no orientation about the proper definition of each comorbidity. As the notification fields can be filled in by any health professional, the understanding about each comorbidity may vary. The last Brazilian survey showed a prevalence of self-reported hypertension of 23.9% in the adult population, while the estimated prevalence of self-reported diabetes is 7.7% [32]. Our data indicated a lower prevalence of comorbidities, with a registered prevalence of 3.4% and 2.6% for chronic cardiovascular diseases and diabetes, respectively, in the studied population. Despite this limitation, data misfiling likely occurred in both groups in the same fashion.

Regarding the socioeconomic status and ethnicity of the patients, although this information could offer additional context for interpreting the results, as these data were missing, they were not included in the analysis. However, it is important to note that since TeleCOVID-MG was a public and free service, most patients supported by this telehealth program did not have private health insurance.

### Conclusions

Despite the massive growth of telehealth initiatives during the pandemic, data regarding the clinical effectiveness of this approach targeting flu syndrome are still lacking [5,6]. This was a retrospective cohort study conducted in a limited-resource region to evaluate the impact of the adoption of a public, structured, multilevel COVID-19 telehealth service for relevant clinical outcomes among adult patients with flu syndrome in comparison with the standard health assistance currently provided, which is primarily delivered in an in-person manner. This study demonstrates that a structured multilevel COVID-19 telehealth service adopted over a large scale can contribute to decreasing the rate of hospital admissions without increasing the mortality and may be considered as a potential and valuable health care strategy, even in regions with limited resources. The success of telehealth initiatives relies on a partnership between the involved stakeholders to coordinate actions within an established health care plan that addresses the specificities of the different target populations.

## Abbreviations

MIS: Mortality Information System
OR: odds ratio
SARS: Severe Acute Respiratory Syndrome
STROBE: Strengthening the Reporting of Observational Studies in Epidemiology
TNMG: Telehealth Network of Minas Gerais
UFMG: Universidade Federal de Minas Gerais

## Data Availability

The data that support the findings of this study are available on request from the corresponding author. The data are not publicly available due to restrictions related to the confidentiality and privacy of the research participants.
